# The Traditional Medicine and Modern Medicine from Natural Products

**DOI:** 10.3390/molecules21050559

**Published:** 2016-04-29

**Authors:** Haidan Yuan, Qianqian Ma, Li Ye, Guangchun Piao

**Affiliations:** 1 College of Pharmacy, Yanbian University, Yanji 133002, China; hdyuan@ybu.edu.cn (H.Y.); qianqian3918@163.com (Q.M.); 2014010621@ybu.edu.cn (L.Y.); 2 Key Laboratory of Natural Resources of Changbai Mountain and Functional Molecules, Ministry of Education, Yanbian University, Yanji 133002, China

**Keywords:** natural products, traditional medicines, drug discovery, traditional uses, chemodiversity

## Abstract

Natural products and traditional medicines are of great importance. Such forms of medicine as traditional Chinese medicine, Ayurveda, Kampo, traditional Korean medicine, and Unani have been practiced in some areas of the world and have blossomed into orderly-regulated systems of medicine. This study aims to review the literature on the relationship among natural products, traditional medicines, and modern medicine, and to explore the possible concepts and methodologies from natural products and traditional medicines to further develop drug discovery. The unique characteristics of theory, application, current role or status, and modern research of eight kinds of traditional medicine systems are summarized in this study. Although only a tiny fraction of the existing plant species have been scientifically researched for bioactivities since 1805, when the first pharmacologically-active compound morphine was isolated from opium, natural products and traditional medicines have already made fruitful contributions for modern medicine. When used to develop new drugs, natural products and traditional medicines have their incomparable advantages, such as abundant clinical experiences, and their unique diversity of chemical structures and biological activities.

## 1. Introduction

Since prehistoric times, humans have used natural products, such as plants, animals, microorganisms, and marine organisms, in medicines to alleviate and treat diseases. According to fossil records, the human use of plants as medicines may be traced back at least 60,000 years [1,2]. The use of natural products as medicines must, of course, have presented a tremendous challenge to early humans. It is highly probable that when seeking food, early humans often consumed poisonous plants, which led to vomiting, diarrhea, coma, or other toxic reactions—perhaps even death. However, in this way, early humans were able to develop knowledge about edible materials and natural medicines [3]. Subsequently, humans invented fire, learned how to make alcohol, developed religions, and made technological breakthroughs, and they learned how to develop new drugs.

Traditional medicines (TMs) make use of natural products and are of great importance. Such forms of medicine as traditional Chinese medicine (TCM), Ayurveda, Kampo, traditional Korean medicine (TKM), and Unani employ natural products and have been practiced all over the world for hundreds or even thousands of years, and they have blossomed into orderly-regulated systems of medicine. In their various forms, they may have certain defects, but they are still a valuable repository of human knowledge [2,4].

In the case of China, Western medicine was introduced in the sixteenth century, but it did not undergo any development until the nineteenth century. Before that, TCM was the dominant form of medical care in the country [5]. Now TCM still plays an important role in China, and it is constantly being developed. TCM is based on 5000 years of medical practice and experience, and is rich in data from “clinical experiments” which guarantee its effectiveness and efficacy. It has developed techniques with respect to such areas as correct dosage, methods of preparing and processing materials, and the appropriate time to collect the various medicinal parts of plants. It is notable that there is increasing convergence between TCM and modern medicine. With the development of modern technology, it has become possible to determine the pharmacology and mechanisms of action of many Chinese herbs, and TCM has become comprehensible in terms of modern medicine [6,7,8,9]. With advances in the theoretical background, therapeutic principles, associated technologies, and understanding of the life sciences, a clearer understanding of the active compounds of TCM has become possible [5].

At the beginning of the nineteenth century, the era of “modern” drugs began. In 1805, the first pharmacologically-active compound morphine was isolated by a young German pharmacist, Friedrich Sertürner, from the opium plant [10,11]. Subsequently, countless active compounds have been separated from natural products. Among them, some follow their traditional uses and the others do not. Later, the development of synthetic techniques led to a significant reduction in the importance of natural products, and there were concerns that the use of some natural products for medicinal purposes might be completely banned. However, natural products are important for the development of new drugs, and these products have been in constant use. Some type of medicines, such as anticancer, antihypertensive, and antimigraine medication, have benefited greatly from natural products [10,12].

The development of new drugs relying purely on modern technology appears to be reaching something of a limit. In developing new drugs, the pharmaceutical industry has tended to adopt high-throughput synthesis and combinatorial chemistry-based drug development since the 1980s; however, the considerable efforts made in this direction have not resulted in the expected drug productivity. Some large pharmaceutical companies are facing great challenges to develop new products. Over the past dozen years, increasing attention has accordingly been paid to natural products in the search for novel drugs in combination with new technology, such as high-throughput selection [13,14].

Natural products, which have evolved over millions of years, have a unique chemical diversity, which results in diversity in their biological activities and drug-like properties. Those products have become one of the most important resources for developing new lead compounds and scaffolds. Natural products will undergo continual use toward meeting the urgent need to develop effective drugs, and they will play a leading role in the discovery of drugs for treating human diseases, especially critical diseases [15].

## 2. Natural Products

Natural products have a wide range of diversity of multi-dimensional chemical structures; in the meantime, the utility of natural products as biological function modifiers has also won considerable attention. Subsequently, they have been successfully employed in the discovery of new drugs and have exerted a far-reaching impact on chemicobiology [16,17,18]. From the past century, the high structural diversity of natural products have been realized from the perspective of physical chemistry. Their efficacy is related to the complexity of their well-organized three-dimensional chemical and steric properties, which offer many advantages in terms of efficiency and selectivity of molecular targets. As a successful example of drug development from natural products, artemisinin and its analogs are presently in wide use for the anti-malaria treatment. This shows how research using natural products has made a significant contribution in drug development [19,20].

Among anticancer drugs approved in the time frame of about 1940–2002, approximately 54% were derived natural products or drugs inspired from knowledge related to such. For instance, the Vinca alkaloids from *Catharanthus roseus*, and the terpene paclitaxel from *Taxus baccata*, are among successful anticancer drugs originally derived from plants [12,21]. During the period between 1981 and 2002, the application of natural products in the development of new drugs—especially in the search for novel chemical structures—showed conspicuous success. In that 22-year time frame, drugs derived from natural products have been significant. That is especially true in the case of antihypertensives, where about 64% of newly-synthesized drugs have their origins in natural product structures [12].

Considering their incomparable chemical diversity and novel mechanisms of action, natural products have continued to play a pivotal role in many drug development and research programs. With time, those natural products have undergone interesting and meaningful developments in their ability to interact with numerous, varied biological targets, and some have become the most important drugs in health care system [14,22,23]. For example, plants, microorganisms, and animals manufacture small molecules, which have played a major role in drug discovery. Among 69 small-molecule new drugs approved from 2005 to 2007 worldwide, 13 were natural products or originated from natural products, which underlines the importance of such products in drug research and development [12,13].

Over the past 50 years, there has been a great diversity of new drugs developed using high-throughput screening methods and combinatorial chemistry; however, natural products and their derived compounds have continued to be highly-important components in pharmacopoeias. Of the reckoned 250,000–500,000 existing plant species, only a tiny proportion has been scientifically researched for bioactivities [13]. Therefore, there is great potential for future discoveries from plants and other natural products which, thus, offer huge potential in deriving useful information about novel chemical structures and their new types of action related to new drug development.

## 3. Traditional Medicines

TM is the oldest form of health care in the world and is used in the prevention, and treatment of physical and mental illnesses. Different societies historically developed various useful healing methods to combat a variety of health- and life-threatening diseases. TM is also variously known as complementary and alternative, or ethnic medicine, and it still plays a key role in many countries today [24,25].

The medicaments used in TM are mostly derived from natural products. In TM, “clinical trials” have been conducted since ancient times. In the case of TCM, considerable experience and advances have been accumulated and developed over the past thousands of years with respect to methods of preparation, selection of herbs, identification of medicinal materials, and the best time for obtaining various different plants. Appropriate processing and dose regulation are urgently needed in TCM to improve drug efficacy and reduce drug toxicity. Considerable amounts of data have been acquired through clinical experiments, and in this way TM has assisted in the development of modern drugs. Through its use of natural products, TM offers merits over other forms of medicine in such areas as the following: discovery of lead compounds and drug candidates; examining drug-like activity; and exploring physicochemical, biochemical, pharmacokinetic, and toxicological characteristics. If any form of TM is applied successfully, it may surprisingly assist in the development of new drugs, thereby resulting in many benefits, such as significant cost reductions.

TCM is now an inseparable part of the Chinese public health system. In recent years, TCM has gradually gained considerable approval as a complementary or alternative medicine in Western countries. Chinese herbal medicine, which is the most important component of TCM, is currently used in the health care of an estimated 1.5 billion people worldwide [26,27]. It should be noted that in TCM, several herbs and ingredients are combined according to strict rules to form prescriptions, which are referred to as formulas (*fang ji* in Chinese). Commonly, a classic formula is composed of four elements—the “monarch”, “minister”, “assistant”, and “servant”—according to their different roles in the formula, each of which consists of one to several drugs. Ideally, these drugs constitute an organic group to produce the desired therapeutic effect and reduce adverse reactions [28].

Kampo is the TM of Japan. Between the fifth and sixth centuries, TCM was introduced to Japan from China; since then, TCM has been significantly altered and adapted by Japanese practitioners to meet their particular circumstances and gradually evolved into Kampo [29]. A recent study has found that some physicians in Japan use Kampo medicines in their daily practice—sometimes as the preferred medication [29,30,31]. Together with radiotherapy or chemotherapy, some Japanese physicians frequently utilize Kampo medicines in treating cancer patients. This indicates how modern Western medicine can be well integrated with TM [30,32]. As the use of Kampo continues to rise in conjunction with Western medicine, there is growing realization of the urgent need to study the interactions between these two types of medicines [28].

Unani is an ancient Greek holistic medical system with a history that can be traced back 2500 years [33]. Since the mid-1970s, when the WHO began to place a greater focus on TM, Unani has attracted considerable attention all over the world, especially in India, where it has been integrated into the national health care system [34].

It was reckoned by WHO that a large quantity of people in the world still depend on TMs for health care [35]. The current status of TM differs in different countries. In 2012, the total value of the TCM industry was equivalent to around one-third of the total for China’s pharmaceutical industry [36]. It has been determined that 80% of the population in Africa makes use of TM—either alone or in conjunction with conventional medicine [37]. By contrast, traditional Aboriginal medicine in Australia is in danger of vanishing owing to the prevalence of conventional medicine [38]. In the case of Israel with its ethnic diversity, modern medicine is prevailing, and TM is declining [39]. Many practitioners of Western medical science think such TM systems as being short of reliability; however, they are adopted by the majority of people in the world [35]. It is possible to produce remarkable synergy and yield great benefits in developing reformed medicines and new drugs by connecting powerful modern scientific techniques and methods with the reasonable ethnobotanical and ethnomedical experiences of TM. Characteristics of several TM systems are summarized in Table 1.

## 4. Drugs Developed from Traditional Medicines that Follow the Traditional Uses

TM is too valuable to be ignored in the research and development of modern drugs. Though it has an enigmatic character, there are also wide contexts for its use in terms of non-Western medical technology or activities. In TM, a single herb or formula may contain many phytochemical constituents, such as alkaloids, terpenoids, flavonoids, *etc.* Generally speaking, these chemicals function alone or in conjunction with one another to produce the desired pharmacological effect [35]. It is notable that a lot of plant-originated drugs in clinical medicine today were derived from TM [21]. In addition, it has been demonstrated that the many valuable drugs derived from plants were discovered through their application in TM [2].

Almost 20 years ago, a thorough investigation of the pharmacopoeias of developed and developing nations and the associated world scientific literature was conducted as part of the WHO’s TM Program. The aim of that study was to determine whether TM really had inspired modern drug discoveries and whether there was any correlation between the current use of various compounds and their application in TM. The study focused on various compounds used in drugs derived from plants in different countries, and it established that TM had indeed played a significant role in developing effective new drugs. That study focused on 122 compounds, 80% of which were found to be related to pharmaceutical effects in folk medicine, and it was determined that these compounds originated from 94 plant species [2].

The acceptability, convenience, and accessibility of TMs have been, and will be, helpful for new drug research [13]. As noted above, artemisinin and other antimalarial drugs are examples of modern drugs based on TMs. Early in China’s Jin Dynasty, Doctor Hong Ge (AD 284–384) recorded the efficacy and related details of *Artemisia annua* L. in treating malaria in his book *Zhou Hou Bei Ji Fang*. That is the earliest record anywhere of treating malaria with *Artemisia annua* L., and it shows that Chinese physicians 1700 years ago had reached a sophisticated level of medical treatment [53,54].

Artemisinin is known as *qinghaosu* in Chinese, and its study has made significant progress, including the synthesis of new artemisinin analogs and derivatives, and research efforts into the biological activities and related mechanisms. As a result, artemisinin, as well as its effective derivatives, are extensively applied throughout the world as new-type anti-malarial drugs [55].

The discovery of artemisinin can be traced back to the 1960s, when tropical malaria was a serious problem during the Vietnam War. North Vietnam requested China to help tackle the malaria problem. The Chinese government approved a project for malaria control and drug research in 1967. The research group made its investigations and carried out a large-scale search of the literature on the subject. As part of the phytochemical and pharmacological research effort, a lot of Chinese herbal medicines were screened and investigated with respect to their toxicity or efficacy. Eventually artemisinin was derived from *Artemisia annua* L. in 1972 [53,55,56]. Artemisinin is quite different from previously-used antimalarial drugs, such as chloroquine, in that it has a novel structure, with a sesquiterpene lactone bearing a peroxy group, and it does not contain nitrogen heterocycles. Compared with previous antimalarial drugs, artemisinin has the merit of high efficiency, quick effect, and low toxicity. Artemisinin is effective in treating various forms of malaria, such as falciparum and cerebral malaria, which are resistant to chloroquine, and its mechanism of action is different from traditional antimalarial drugs. The discovery of artemisinin was a great success for TCM at a special period in China’s history, and it was achieved through a well-organized team of hundreds of researchers [56]. Since that breakthrough, scientists have conducted comprehensive research in such areas as pharmaceutical chemistry, organic synthetic chemistry, and chemical biology. Through etherification and esterification, they have produced a series of well-known new drugs, such as artemether and artesunate. Those drugs have improved efficacy and solubility, which are of benefit for patients receiving oral or intravenous administration and have overcome the high parasite recrudescence rate and low solubility of artemisinin [55,56,57]. Most importantly, one of these scientists, Youyou Tu, was just awarded the 2015 Nobel Medicine Prize for her significant devotion in discovering artemisinin.

The discovery of artemisinin illustrates how TCM constitutes a great store of knowledge about natural products, such as Chinese herbs, and holds much future promise. The discovery of successful new drugs can proceed by profiting from this knowledge [56]. Some drugs or compounds isolated from Chinese herbal medicines which follow the ethnomedical uses are summarized in Table 2.

## 5. Drugs Developed from Natural Products

In clinical practice in China in the 1960s, it was found that *Schisandra chinensis* (Turcz.) Baill.—a traditional Chinese herb—had obvious enzyme-reducing and hepatoprotective effects. Chinese scientists then began isolating the chemical constituents of *S. chinensis*. In the subsequent total chemical synthesis and pharmacodynamic study of schisandrin C (which is one of the compounds of *S. chinensis*), researchers found that the intermediate compound bifendate had a stronger pharmacological activity and that the cost of preparation was low. They discovered that it may be used to lower the enzyme content in the treatment of hepatitis B virus [57].

Since the end of the 1980s, chemists and pharmacologists at the Chinese Academy of Medical Sciences have been closely cooperating in studying the structure and activity relationships of bifendate and its analogs. As part of their research, a series of novel derivatives were synthesized. After screening using a number of chemical and pharmaceutical liver injury models, it was found that the hepatoprotective activities of the derivatives were closely related to the locations of dimethylenedioxy in two benzene rings, the length of the side-chain carboxylic acid, and the heterocycle between the two benzene rings. Finally, a new compound, bicyclol—formulated as 4,4″-dimethoxy-5,6,5′,6′-bis(methylene-dioxy)-2-hydroxy-methyl-2′-methoxycarbonyl biphenyl—was designed and synthesized. Bicyclol had greater *in vivo* absorption, and better bioavailability and biological activity, than bifendate owing to the introduction of the 6-hydroxymethyl group and 6′-carbomethoxy in the side chain [72]. Pharmacological results of bicyclol showed antifibrotic and hepatoprotective effects against liver injury and liver fibrosis induced by CCl_4_ or other hepatotoxins in mice and rats; it also exhibited the antihepatitis virus effect in the 2.2.15 cell line and duck model with viral hepatitis [73,74].

In clinical trials, it was found that the increased levels of serum alanine aminotransferase and aspartate aminotransferase were dramatically decreased by bicyclol. It was also found that bicyclol prohibited hepatitis B virus replication in chronic hepatitis B patients [75]. Compared with previous anti-hepatitis drugs, bicyclol exhibited a more consolidated effect after the drug was discontinued; the rebound rate was low, with fewer adverse reactions and higher oral bioavailability [76]. Based on previous studies in such areas as synthesis, pharmacology, toxicology, pharmacokinetics, preparation, and quality control, researchers determined that the new antihepatitis drug bicyclol offered significant hepatoprotective effects, antihepatitis virus activity, and fewer adverse reactions [57]. Bicyclol has been approved for the treatment of chronic viral hepatitis in China since 2004 [73]. Bicyclol has independent intellectual property rights and belongs to Class 1 of China’s New Chemical Drug. The drug is one of the anti-inflammatory and hepatoprotective drugs recommended by the “Guidelines on Liver Disease Clinical Diagnosis and Treatment” in China, and it has been exported to many countries [57,76].

In the same decade in which Chinese scientists found that *S. chinensis* (Turcz.) Baill. had obvious enzyme-reducing and hepatoprotective effects, a program screening for cancer drugs from plants began in 1960 at the National Cancer Institute in the United States. Neither China nor the United States knew what the other was doing in this area. In that US project, 650 plant samples were gathered in three states. After the initial cytotoxicity tests were carried out using crude extracts, *Taxus brevifolia* was chosen for further research.

Taxol was isolated as a new compound from *T. Brevifolia*. Taxol has an unusual chemical structure and radically distinctive mechanism of action and was developed as a novel anticancer drug in subsequent decades. Nevertheless, the drug attracted little attention during the early stage of its development because of its poor solubility in water, low yield from natural products, and other disadvantages, particularly by the medical society. The story of Taxol involved many events that nearly resulted in discontinuation of the research. Fortunately, it underwent extraction, isolation, and structural determination; its activity against solid tumors and its mechanism of action were established, and it became developed for clinical practice. Finally, Taxol was approved by the US Food and Drug Administration for treating ovarian cancer in 1992—21 years after the initial breakthrough paper recording its isolation and structural identification. Taxol has remained a basic drug for treating various forms of cancer, and is still being used to develop new synergistic groups of anticancer drugs [77,78,79]. Some drugs or compounds isolated or developed from natural products are summarized in Table 3.

## 6. Discussion

Human history is also the history of medicines used to treat and prevent various diseases. To counter the danger from serious illnesses and to guarantee survival of the species, it is necessary to continually produce better drugs. With time, the use of these natural products as TM increased. Modern medicine has benefited considerably from TM in two areas: drugs with similar effects and drugs with different effects from those of TM. From the history of drug development, it is evident that many drugs have been derived as a result of inspiration from TM.

The application of, and research into, natural products are far from satisfactory. A number of problems need to be addressed in the future. For example, synergistic effects may exist among the compounds that occur in natural products; however, the modes and mechanisms of action are seldom very clear. It is, therefore, necessary to make full use of such synergetic effects toward improving the effectiveness of drugs. However, it is also requisite that any adverse effects of natural products be properly reduced to meet safety standards.

With the riches of modern technology, such as in synthesis, fermentation, pharmacology, pharmacodynamics—together with biological diversity, chemodiversity, and great breakthroughs in evolutionary techniques or concepts—combined with a wealth of knowledge about natural products, it will be possible to establish a large compound library for drug screening [89]. This will enhance the possibilities for individual treatment and prevention of disease. Humankind needs to learn more from natural products and traditional medicines.

In order to further promote the development of modern medical research on natural products, humans have to face up to various difficulties and challenges. Valuable information on natural products and TMs is mixed in a large number of documents, data, and useless rumors. Furthermore, one plant or formula of natural products and TMs contains a large number of chemical constituents, including active, invalid, and possible synergistic components. Therefore, great effort should be made at first to remove the dross and take the essence—precious experience of natural products and TMs. Furthermore, in many cases, the role of single compound from natural products and TMs is paid much attention to. However, as a matter of fact, one advantage of TM’s therapeutics is the “synergism”; that is, often multiple components in TMs play a synergistic role which is greater than that of the individual drug. In the meantime, the “1 disease, 1 target, 1 drug” mode cannot treat some complex diseases effectively, such as cardiovascular disease and diabetes. Thus, the treatment has seen a shift to the “multi-drugs and multi-targets” mode for combination therapies. Therefore, in the future, multidisciplinary collaborative research, closely cooperated with new ideas, such as network pharmacology and big data, will be possible to explain the synergism and other mechanisms of natural products and TMs from which more and better new drugs and treatment will be discovered and inspired.

## Figures and Tables

**Table 1:** Characteristics of several important traditional medicine systems.

Name	Origin and Developing Nation	Characteristics of Theory or Application	Current Role or Status	Modern Research
Traditional Chinese medicine (TCM) [26,28,40,41,42,43]	China Thousands of years ago.	TCM is based on Yinyang and Wuxing concepts. A TCM formula includes a group of various drugs that function together congenially to achieve a synergistic effect. A classic formula is composed of four elements: monarch, minister, assistant, and servant according to their roles in the formula.	Both TCM and conventional medicine exist at every gradation of the health-care system, and both are covered under public and private insurance. There is a TCM division in most ordinary hospitals and TCM services are supplied for both inpatients and outpatients. TCM is attracting increasing attention, interest, and acceptance around the world.	The pharmacology of TCM has made great advancements. In recent decades, many TCM active compounds and compound-based therapeutics have been discovered. Great efforts have been made to reveal the underlying molecular mechanisms of TCM.
Ayurveda [35,44]	India Ayurveda can be dated back to the pre-Vedic epochs (4000 BC–1500 BC)	Ayurveda uses natural elements to eradicate the main cause of the disease by reinstating balance. The Ayurvedic philosophy is to live a healthy life to avoid the appearance of imbalance and unnecessary pain. In many Ayurvedic treatments, multiple herbs are united in a special quotient to create an ideal therapeutic effect and lessen the toxicity.	More than 400,000 Ayurveda practitioners are registered. The Indian government has an official body to ensure Ayurveda’s educational efforts, quality, and practice.	Pharmacologically-active compounds of Ayurvedic medicine and their effectiveness in treatment has been increasingly recognized.
Unani medicine [33,34,45,46]	India Unani medicine derived from Greco-Arabic medicine dating back 2500 years and developed during the Arab civilization.	It treats a person’s body, mind, and soul as a whole. Unani looks upon the human body as a single unit, which consists of four basic elements which have four disparate temperaments respectively. A person’s temperament reflects their physical characteristics and natural disposition. Disproportion in temperament makes the human body susceptible to many illnesses.	Unani is accepted by India as meeting the health-care needs of people and has gained formal status. Unani has been acknowledged by the WHO as an alternative health-care system. Unani is one of the most important traditional medicine systems.	Many bioactive ingredients have been separated from mangrove plants which are used in Unani medicine.
Kampo (traditional Japanese medicine) [30,47]	Japan Kampo was introduced from China via the Korean peninsula in the 5th or 6th century.	Kampo was developed over the past 1400 years and has been organically unified with Japanese original therapies. Kampo treats every human being as a complete and self-controlled whole in which body and mind impact mutually. Diseases are thought to originate from the disorders of psyche and soma and herbals are trusted to affect the soul and the body equally. Kampo therapy places emphasis on the sufferer as a whole instead of on the illness.	Kampo is incorporated into the health-care system in Japan. All citizens can use of Kampo herbal formulas approved by the government.	Kampo formulas are produced by certificated drug firms under strict quality management standards. Both the government and drug firms are deeply involved in surveillance of all processes to ensure the quality and safety of Kampo formulas. There has also been a focus on examining the efficacy of Kampo formulas and exploring related mechanisms. Kampo is regarded as very safe.
Traditional Korean medicine (TKM), Sasang constitutional medicine (SCM) [42,48,49,50]	SCM is a division of Korean traditional medicine. It was first introduced in the mid-19th century.	SCM classifies persons into four Sasang types: Tae-Yang, So-Yang, Tae-Eum, and So-Eum according to his/her inborn features. SCM is holistic. SCM is theoretically similar to personalized medicine. SCM supplies individualized and constitution-specific treatments for various problems.	Although the conventional health-care organization is quite good in Korea, 86% of people still employ SCM. Traditional medicine doctors can supply Korean SCM both in private and public hospitals. Both national medical insurance and private insurance cover Korean SCM services.	The Lee Jema project to supply scientific proof of SCM began in 2006 and is supported by the Korean government. It has gained many significant achievements involving constitution-diagnostic means, constitution-specific disease vulnerabilities, and genetic research.
Traditional Aboriginal medicine [38,50]	Australia	Indigenous peoples of Australia believe that health problems have three types of causes: natural bodily causes, harmful spirits, or witchcraft.	Currently, there is only one national folk organization in operation. During 2010–2011, 32.1% of the chief, indigenous health-care organizations in Australia provided some kind of traditional medicine services. Because of colonization, traditional Aboriginal medicine is in danger of becoming extinct.	
Traditional medicine in Africa [25,37,38,51]	Africa	Traditional medicine doctors treat patients holistically. They generally seek to recombine the mental and social equipoise of sufferers according to social relationships and rules. The accessibility of traditional medicine is one of the most important reasons for its popularity across Africa. Traditional medicine exemplifies respect for the cultural heritage.	Eighty percent of African people use traditional medicine either by itself or with conventional medicine. Up to 80% of Ghanaians and Ethiopians depend on traditional medicine for their main health-care demands. Ghana’s traditional medical system has been integrated into the national health-care system and, therefore, it is comparatively well organized.	Research on *Hydnora africana*, which is used as ethnomedicine in Africa, has demonstrated the antioxidant and antibacterial activities of natural products.
Russian herbal medicine [52]	Russia 10th century	Due to the special geographical environment of Russia, Russian herbal therapy has collected and adopted traditional medicine methods that were introduced from Europe and Asia. The Russian Federation follows the State Pharmacopoeia of the USSR; 32 of 83 individual plant monographs are found only in this Pharmacopoeia.	Herbal therapy is a formal and independent department of medicine in Russia; thus, herbal medicinal products are regarded as official remedies. A recent survey shows that 14% of the Russian people frequently use herbal remedies and 44% use them occasionally.	Soviet/Russian researchers have focused mainly on the development of adaptogens derived from plants. The collection of plants with expectorant effects shows huge potential.

**Table 2:** Some drugs or compounds isolated from Chinese herbal medicines which follow the traditional uses.

Plant Origin	Drugs or Compounds	Chemical Structures	Effects or Indications	Ancient Chinese Literature Recording Chinese Herbal Medicines with Same Effects and the Published Time
*Artemisia annua* L*.* [53,55]	Artemisinin	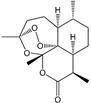	Anti-malarial	Zhou Hou Bei Ji Fang (Jin Danasty, AD 266–420)
*Corydalis yanhusuo* W.T.Wang [58,59]	Tetrahydropalmatine	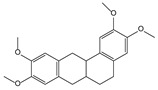	Analgesic	Lei Gong Pao Zhi Lun (Nanchao Song Dynasty, AD 420–479)
*Ligusticum chuanxiong* Hort. [60]	Tetramethyl-pyrazine	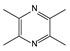	Mmyocardial ischemia-reperfusion injury	Shen Nong Ben Cao Jing (Donghan Dynasty, AD 25–220)
*Paeonia lactiflora* Pall. [61,62]	Paeoniflorin	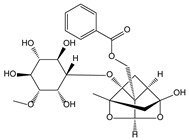	Analgesic	Shen Nong Ben Cao Jing (Donghan Dynasty, AD 25–220)
*Epimedium brevicornum* Maxim. [63,64]	Icariin	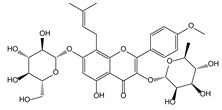	Osteoporosis	Shen Nong Ben Cao Jing (Donghan Dynasty, AD 25–220)
*Pueraria lobata* (Willd.) Ohwi [65]	Puerarin	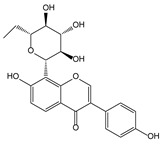	Diabetes	Shen Nong Ben Cao Jing (Donghan Dynasty, AD 25–220)
*Salvia miltiorrhiza* Bunge [66,67]	Salvianolic acid B	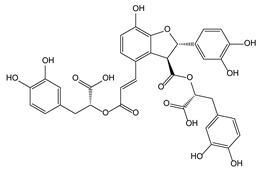	Cardiovascular and cerebrovascular diseases	Shen Nong Ben Cao Jing (Donghan Dynasty, AD 25–220)
*Uncaria rhynchophylla* (Miq.) Jacks. [68]	Rhynchophy-lline	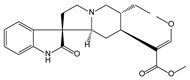	Antihypertensive	Ming Yi Bie Lu (Nanchao Liang Dynasty, AD 502–557)
*Saussurea lappa* (Decne.) C.B. Clarke [69]	Costunolide	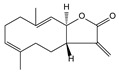	Anti-gastric ulcer, antispasmodic	Shen Nong Ben Cao Jing (Donghan Dynasty, AD 25–220)
*Gastrodia dlata* Bl. [70,71]	Gastrodin	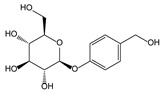	Anti-convulsion, analgesic	Shen Nong Ben Cao Jing (Donghan Dynasty, AD 25–220)

**Table 3:** Some drugs or compounds isolated or developed from natural products.

Origin (Plant, *etc.*)	Drugs or Compounds	Chemical Structures	Effects or Indication
*Schisandra chinensis* (Turcz.) Baill. [55,72,73,74,75,76]	Schisandrin C, bicyclol, bifendate	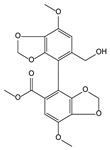	Hepatoprotective, anti-hepatitis B virus
		bicyclol	
*Taxus brevifolia* [77,78,79,80]	Taxol, docetaxel	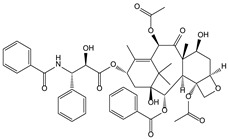	Antitumor
		taxol	
*Aspergillus terreus* [81]	Lovastatin	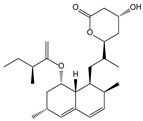	Hyperlipoidemia
*Camptotheca acuminata* Decne. [1]	Camptothecin, irinotecan and topotecan	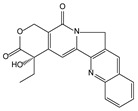	Antitumor
		camptothecin	
*Gimkgo biloba* L. [82]	Ginkgolide B	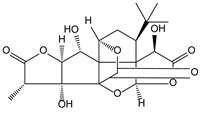	Cerebral infarction
*Polygonum multiflorum* Thunb. [83]	Stilbene glycoside	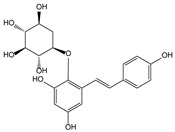	Vascular dementia
*Ranunculus ternatus* hunb. [84,85]	Ternatolide	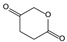	Anti-tuberculosis
*Curcuma longa* L. [86]	Curcumin	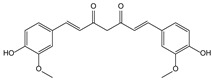	Hypolipidemic
*Ophiopogon japonicus* (L.f.) Ker-Gawl. [87]	Polysaccharide MDG-1	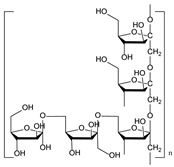	Anti-myocardial cell injury
*Chromobacterium violaceum* [88]	Romidepsin	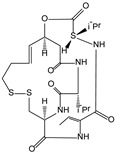	Antitumor
